# Surgical outcomes and factors associated with postoperative complications of colorectal cancer in a Colombian Caribbean population: Results from a regional referral hospital

**DOI:** 10.1002/cnr2.1766

**Published:** 2022-12-20

**Authors:** Edgar Ernesto Vergara Dagobeth, Gian Alberto Núñez Rojas, Juan Carlos Hoyos Valdelamar, Ivan David Lozada‐Martínez, Amileth Suarez Causado, Alexis Rafael Narvaez‐Rojas

**Affiliations:** ^1^ School of Medicine Universidad de Sucre Sincelejo Colombia; ^2^ Department of Surgery, School of Medicine Universidad de Cartagena Cartagena Colombia; ^3^ Grupo Prometeus y Biomedicina Aplicada a las Ciencias Clínicas School of Medicine, Universidad de Cartagena Cartagena Colombia; ^4^ Medical and Surgical Research Center Future Surgeons Chapter, Colombian Surgery Association Bogotá Colombia; ^5^ International Coalition on Surgical Research Universidad Nacional Autónoma de Nicaragua Managua Nicaragua

**Keywords:** colonic neoplasms, mortality, postoperative complications, rectal neoplasms

## Abstract

**Introduction:**

Colorectal cancer is the most common malignant neoplasm of the gastrointestinal tract. Its incidence and mortality vary markedly at a global level. Assessing the epidemiological behavior of this condition allows reevaluating diagnostic, therapeutic and prognostic options, based on new findings. In Colombia, few studies have correlated variables associated with surgical and oncological outcomes in this type of cancer. Then, the aim of this study was to evaluate the surgical outcomes and factors associated with postoperative complications of colorectal cancer in a Colombian Caribbean Population.

**Methods:**

Retrospective cross‐sectional study, including patients with a histopathological diagnosis of colorectal cancer who underwent open or laparoscopic surgery, during a period of two years (2018–2020), from a regional referral hospital. Clinical history variables were collected. Frequencies and prevalence ratios were calculated.

**Results:**

A total of 84 patients were finally included. Adenocarcinoma of non‐special type with advanced clinical stages was the most prevalent (72.6%). Rectal neoplasia (45.2%) was the most frequent anatomical subsite, followed by proximal colon (*p* = 0.026). The anatomical subsite of the neoplasm, intraoperative complication (PR 1.38; 95% CI, 1.21–1.59, *p* = 0.001) and intensive care stay (PR 1.062; 95% CI, 1.01–1.12, *p* = 0.048) were associated with postoperative outcome.

**Conclusions:**

The anatomical subsite of the neoplasm location, the presence of intraoperative complications and the stay in intensive care may be associated with the surgical and oncological outcome of individuals with colon cancer from the Colombian Caribbean region.

## 1 INTRODUCTION

Colorectal cancer is the most common malignant neoplasm of the gastrointestinal tract. Its incidence and mortality vary considerably at a global level. According to the GLOBOCAN data, by 2020, colorectal cancer was the second most frequent (1 931 590 cases ‐ 10% of total cases) and the second most deadly (935 173 cases—9.4% of total number of deaths), below lung cancer.^1^ Similarly, in Colombia, colorectal cancer is the third most frequent cancer in both sexes, being surpassed by breast and prostate cancer.^1^ It is the second most frequent cancer in women with an incidence of 16.7 cases per 100 000 women and 17.3 cases per 100 000 men.^1^ This represented approximately 10% of the total number of new cancer cases in Colombia.^1^ In Cartagena, the local health analysis carried out in 2019 found that neoplasms ranked third in frequency of cancer mortality,^2^ making them one of the public health priorities.

Surgical treatment is the most common treatment for resectable colorectal cancer, and during the last decades, it has presented great improvements in terms of preoperative evaluation, instruments, surgical techniques, intraoperative monitoring and postoperative care.^3^ Laparoscopic surgery, as opposed to open surgery, is recommended in patients without obstruction, perforation or local invasion. A consensus study published a few years ago by McNair et al,^4^ identified important perioperative domains to evaluate in colon and rectal oncological surgery.^4^ Oncological outcomes included long‐term survival, cancer recurrence and resection margins, and operative outcomes included anastomotic leakage, perioperative survival, surgical site infection, stoma‐related complications, and conversion to open procedure.^4^ The authors concluded that such domains must be constantly evaluated and taken into account in order to define interventions based on the behavior of populations. In Colombia, few studies have correlated variables associated with surgical and oncological outcomes in this type of cancer,^5^ and among the objectives of global surgery,^6^ the need to produce evidence on the outcomes and progress of oncological surgery in the treatment of cancer has been highlighted, in order to control the burden of this disease, reduce health costs and catastrophic expenses, especially in low‐and middle‐income countries, such as Colombia.^7^, ^8^

The Colombian Caribbean region is one of the regions with the greatest inequality and difficulties in access to health care, which influences the general outcomes described in the literature on colorectal cancer. Currently, there is no evidence that has studied the relationship of surgical and oncological behavior with colorectal cancer outcomes in this region, preventing the application of in‐hospital and out‐of‐hospital strategies to improve the quality of care and outcomes. Studies currently underway seek to describe the correlation between genotypic and phenotypic expression of colorectal cancer in this region, which is mainly composed of Afro‐descendant, mixed race and indigenous populations, in order to define the safest and most effective interventions for the control and resolution of this disease. Taking into account this gap in evidence, and the importance of knowing these outcomes to correlate them with findings in the molecular and genetic study of colorectal cancer in this particular population, the aim of this study was to evaluate the surgical outcomes and factors associated with postoperative complications of colorectal cancer in a Colombian Caribbean Population.

## 2 METHODS

### 2.1 Study design and participants

A retrospective cross‐sectional study was carried out, evaluating the surgical outcomes and factors associated with postoperative complications of patients with colorectal cancer who underwent surgery in a regional tertiary center, by analyzing the medical records. This is a regional referral center that receives patients from the Caribbean region, especially those with limited resources. Annually, the gastroenterology surgery unit has historically received approximately 30 cases of colorectal cancer that need to be managed surgically. In the study period (January 2018–February 2020), a total of 96 patients were received.

Patients were included if they met all the following criteria: (1) had been operated on by the surgeons of the gastrointestinal surgery unit; (2) had a definitive diagnosis of cancer and classification by histopathological study; (3) had a complete medical history, for the analysis of clinical data; and (4) were 18 years of age or older. Patients who had been previously operated for the same cause, who had incomplete data and those who came from a region other than the Colombian Caribbean region were excluded in order to avoid discrepancies in the clinical, oncologic and epigenetic behavior of colorectal cancer in the region. After the application of inclusion and exclusion criteria, 84 patients were finally included.

### 2.2 Data extraction

Information was collected on clinical variables such as age, sex, type of surgical procedure schedule, lesion location, anatomical subtype, pre‐surgical anesthetic classification ASA (American Society of Anesthesiologists),^9^ preoperative laboratory variables (hemoglobin, albumin, carcinoembryonic antigen), variables related to the surgical procedure (approach, type of procedure, duration of surgery, initiation of the oral route, presence of intraoperative complication, days of hospitalization) and postoperative variables (postoperative mortality, postoperative carcinoembryonic antigen), including presence of postoperative complication within the hospital stay related to the surgical procedure. In addition, information was collected on histopathological variables such as relapse, margin compromise, extent of surgery, histological type, histological grade, resected lymph nodes, tumor diameter, tumor staging classification (TNM), clinical stage and variables related to complementary treatment (chemotherapy, radiotherapy).

### 2.3 Outcomes assessed

The primary endpoints evaluated were: postoperative complication (defined as the appearance of local or systemic symptoms associated with the alteration of the normal evolution of the patient after surgery), recovery time (defined as the time from the surgical procedure to the restoration of the patient's functional capacity [e.g., initiation of the oral route, among others]), postoperative morbidity (defined as the occurrence of significant morbidity, with the need for intensive care admission or longer hospital stay compared to the average) and mortality (defined as death between the time of the surgical procedure, until hospital discharge).^4^, ^10^ The secondary endpoints were the factors that could predict complications and mortality.

### 2.4 Statistical analysis

Quantitative variables were measures of central tendency type average (x̄ or Median [Me]) with their respective measures of dispersion standard deviation (SD) and interquartile range (IQR) depending on the normality of their distribution, which was calculated using the Shapiro–Wilk test. The descriptive analysis of qualitative variables was performed by calculating absolute and relative frequencies. For the comparison of the distribution of postoperative complications for clinical, surgical procedure‐related and postoperative variables, the chi‐square test or the Mann Whitney test was used for qualitative variables, depending on the result of the normality test; for the comparison of distributions of quantitative variables, the ANOVA test or the Kruskall–Wallis test was used, depending on the distribution of the variable.

In addition, a Poisson regression analysis was performed to determine the prevalence ratios (PR) associated with the significant variables in the bivariate analysis, with the presence of postoperative complications as the dependent variable. An analysis was performed controlling for possible confounding variables. Values of *p* < 0.05 were taken as significant. Data analysis was performed with the Stata software version 16.0 (StataCorp, Texas, USA).

### 2.5 ETHICS STATEMENT

This study was approved by the ethics committee of the institutions, complied with the Declaration of Helsinki,^11^ and was classified in the category of research without risk, according to article 11 of Resolution 8430 of 1993 of the Colombian Ministry of Health.^12^ All patients or their relatives signed the informed consent form. The management of the data obtained from the medical records was carried out in accordance with the provisions of Law 23 of 1981 of the Colombian Congress^13^ and Resolution 1995 of 1999 of the Colombian Ministry of Health.^14^

## 3 RESULTS

A total of 84 patients were included, who underwent surgery for a diagnosis of colorectal cancer. The mean age was 59.5 ± 17.1 years. 45.2% (*n* = 38) of the patients were male and 51.1% (*n* = 43) of the procedures were performed on an emergency basis. The most frequent lesion location was the rectum with 45.2% (*n* = 38), followed by the sigmoid and ascending colon (16.6% and 11.9%, respectively). According to the ASA classification, the majority were categorized as ASA III (51.1%, *n* = 43). It was found that 52.1% (*n* = 43) had hemoglobin values higher than 11 g/dl. Likewise, 55.9% (*n* = 47) of the patients had albumin values between 3.1 and 3.5 mg/dl. About 50% of the patients included had no carcinoembryonic antigen (CEA) report, while 33.3% (*n* = 28) obtained results greater than 5 μg/L **(**Table 1 **)**.

**TABLE 1:** Description of preoperative and clinical variables of the study population.

Variable	x̄ ± SD / Me (IQR)	N (%)	*p‐value*
Age	59.5 ± 17.1		0.06
Sex			0.43
Male		38 (45.2)	
Female		46 (54.7)	
Type of surgery			0.27
Emergency		43 (51.1)	
Programmed		41 (48.8)	
Localization			0.04
Appendix		2 (2.3)	0.08
Cecum		7 (8.3)	
Ascending colon		10 (11.9)	
Transverse colon		6 (7.1)	
Descending colon		7 (8.3)	
Sigmoid colon		14 (16.6)	
Rectum		38 (45.2)	
Anatomical subtype			0.02
Proximal colon		25 (29.7)	
Distal colon		21 (25.0)	
Rectum		38 (45.2)	
ASA			0.25
I		12 (14.2)	
II		43 (51.1)	
III		28 (33.3)	
IV		1 (1.1)	
Preoperative hemoglobin			0.78
<9		8 (9.5)	
9–11		33 (39.2)	
>11		43 (52.1)	
Preoperative albumin			0.94
2–2.5		3 (3.5)	
2.6–3		12 (14.2)	
3.1–3.5		47 (55.9)	
>3.5		22 (26.1)	
Preoperative CEA			0.47
No report		42 (50.0)	
<5		14 (16.6)	
>5		28 (33.3)	

A total of 73.8% (*n* = 62) of the patients underwent laparoscopic surgery. No conversions from laparoscopic to open approach were performed. The most common type of procedure was right hemicolectomy (26.1%), followed by colostomy (20.2%). The mean operative time was 159.4 ± 50.5 min, with the average duration in patients who underwent open approach surgery being 172.2 ± 43.7 min, and 154.9 ± 53.3 min in patients who underwent laparoscopic surgery. The vast majority of patients had no intraoperative complications (96.4%); and among those with complications, one case was a vascular complication, one was a cardiopulmonary complication, and one case of complication was classified under other causes **(**Table 2 **)**.

**TABLE 2:** Description of variables related to the surgical procedure.

Variable	x̄ ± SD / Me (IQR)	N (%)	*p‐value*
Type of approach			0.09
Open		22 (26.1)	
Laparoscopy		62 (73.8)	
Type of surgery			0.02
Right hemicolectomy		22 (26.1)	
Left hemicolectomy		14 (16.6)	
Sigmoidectomy		5 (5.9)	
Transversectomy		2 (2.3)	
Total colectomy		2 (2.3)	
Abdominoperineal resection		3 (3.5)	
Low anterior resection		14 (16.6)	
Ultra‐low anterior resection		5 (5.9)	
Colostomy		17 (20.2)	
Surgical time	159.4 ± 50.5 min		0.17
Intraoperative complication			
Without complication		81 (96.4)	0.01
Vascular type		1 (1.1)	
Cardiopulmonary type		1 (1.1)	
Other		1 (1.1)	
Start via oral route	58.2 ± 30.2 h		0.04
<12 hours		1 (1.1)	0.06
12–48 hours		22 (18.4)	
>48 h		61 (72.6)	
Postoperative complication			
Without complication		61 (72.6)	
Operative site infection		9 (10.7)	
Metabolic		1 (1.1)	
Cardiopulmonary		4 (4.7)	
Anastomotic dehiscence		2 (2.3)	
Perforation		4 (4.7)	
Hemorrhage		2 (2.3)	
Other		1 (1.1)	
Hospitalization	5 (4–7) days		0.01
Postoperative mortality			0.01
Non‐mortality		71 (84.5)	
Hemorrhage		1 (1.1)	
Septic shock		7 (8.3)	
Cardiopulmonary		4 (4.7)	
Other		1 (1.1)	

The mean time to initiation of the oral route was 58.2 ± 30.2 hours. Most of the patients started the oral route after 48 hours of the procedure (72.6%); the mean time to start the oral route in patients with open surgery was 69.8 ± 36.0 hours, while for patients with laparoscopic surgery the result was 54.4 ± 27.3 hours. 72.6% (n = 61) of the patients had no postoperative complications. The most frequent complication in the population was surgical site infection (10.7%), followed by perforation and cardiopulmonary complication (both with 4.7%) and anastomotic dehiscence (2.3%) in only two cases. The median hospitalization time was 5 days (IQR 4–7 days). Excluding patients without complications, it was observed that patients who underwent laparoscopic approach had a median hospitalization time of 4.64 ± 2 days, while patients with open approach had a median hospitalization time of 7.6 ± 8.12 days.

Most of the cases in which the patient presented with surgical site infection occurred in cases of emergency surgery and in those who underwent right hemicolectomy. Postoperative mortality within the hospitalization related to the procedure was 15.5% (*n* = 13), the most frequent cause being septic shock with 8.3% (*n* = 7), followed by cardiopulmonary causes with 4.7% (*n* = 4).

About 5.9% (*n* = 5) of the patients presented CEA values less than 5 μg/L. 19.0% (*n* = 16) had local–regional relapse, while 13.1% (n = 11) had proximal margin involvement. About 41.6% (*n* = 35) of the patients presented surgery without residual extension, while 22.6% (*n* = 19) presented microscopic extension and (15.4%) presented macroscopic extension **(**Table 3 **)**. The most commonly reported type of neoplasm was adenocarcinoma (72.6%, *n* = 61) with moderate histological differentiation (39.2%, *n* = 33). The relationship between pathological features of the disease such as stage and histopathological diagnosis with tumor location is described in Table 4.

**TABLE 3:** Distribution of postoperative variables evaluated in the study population.

Variable	x̄ ± SD / Me (IQR)	N (%)	*p‐value*
Postoperative CEA			0.04
*No report*		76 (90.4)	
<5		5 (5.9)	
5–15		1 (1.1)	0.08
>15		1 (1.1)	
Local‐regional relapse		16 (19.0)	0.13
*Proximal margin involvement*			0.24
No		56 (66)	
Yes		11 (13.1)	
No report		17 (20.2)	
*Distal margin involvement*			0.29
No		54 (64.2)	
Yes		2 (2.3)	
No report		28 (33.3)	
*Radial margin involvement*			0.07
No		37 (44.0)	
Yes		17 (20.2)	
No report		30 (35.7)	
*Surgical extension*			0.15
No residual extension		35 (41.6)	
Microscopic extension		19 (22.6)	
Micro and macroscopic extension		13 (15.4)	
No report		17 (20.2)	
*Histological type*			0.62
Adenocarcinoma		61 (72.6)	
Mucinous adenocarcinoma		11 (13.1)	
Medullary adenocarcinoma		5 (5.9)	
Undifferentiated carcinoma		3 (3.5)	
Neuroendocrine carcinoma		4 (4.7)	
*Histological grade*			0.67
Well differentiated		33 (39.2)	
Moderately differentiated		41 (48.8)	
Poorly differentiated		8 (9.52)	
Resected lymph nodes	6 (1–10)		0.08
Invaded nodes	0 (0–1)		0.70
Nodal index	0 (0–0.15)		0.85
Tumor diameter	47.1 ± 21.9 mm		0.52
Tumor thickness	35 (22–45) mm		0.44
*TNM*			0.18
0		3 (3.5)	
I		10 (11.9)	
IIA		6 (7.1)	
IIB		9 (10.7)	
IIC		10 (11.9)	
IIIA		9 (10.7)	
IIIB		13 (15.4)	
IIIC		11 (13.1)	
IVA		8 (9.5)	
IVB		5 (5.9)	
*Metastasis*			
Liver		8 (9.5)	
Lung		2 (2.3)	
Other		2 (2.3)	
Clinical stage			0.51
Local disease		39 (46.4)	
Local‐regional disease		33 (39.2)	
Systemic or metastatic disease		12 (14.2)	
*Complementary treatment*			
Chemotherapy		24 (28.5)	0.01
Radiotherapy		26 (30.9)	0.01
Biological therapy		4 (4.7)	0.91

**TABLE 4:** Relationship between location, clinical stage and histological type of tumor.

Variable	Proximal colon	Distal colon	Rectum
	*N* (%)		
*Clinical stage*			
Local disease	11 (13.1)	13 (15.4)	1 (1.1)
Local‐regional disease	14 (16.6)	6 (7.1)	1 (1.1)
Systemic or metastatic disease	14 (16.6)	14 (16.6)	10 (11.9)
*Histological type*			
Adenocarcinoma	17 (20.2)	13 (15.4)	31 (36.9)
Mucinous adenocarcinoma	5 (5.9)	3 (3.5)	3 (3.5)
Signet ring cell adenocarcinoma	3 (3.5)	2 (2.3)	0 (0)
Undifferentiated carcinoma	0 (0)	0 (0)	3 (3.5)
Neuroendocrine carcinoma	0 (0)	3 (3.5)	1 (1.1)

The mean tumor diameter was 47.1 ± 21.9 mm, with a median thickness of 35 (IQR 22–45 mm). Regarding the distribution of stages by TNM, it was found that the majority of patients were in stage III (39.2%, *n* = 33). The organ most frequently involved by metastasis was the liver with 9.5% (*n* = 8). However, 85.1% (*n* = 72) of the patients did not present metastases. About 14.2% (*n* = 12) of the included patients had systemic disease, while 46.2% (*n* = 39) had local disease. When relating the type of procedure to the clinical stage, it was found that of the 17 patients who underwent colostomy, 2 were classified as stage I, 9 as stage II and 6 as stage III. About 28.5% (*n* = 24) of the included patients received neoadjuvant chemotherapy, 30.9% (*n* = 26) radiotherapy and 4.5% (*n* = 4) biological therapy. No patient who received adjuvant or neoadjuvant treatment presented postoperative complications. The presence or absence of postoperative complications according to the type of surgical approach is described in Table 5, and the characteristics of clinically important variables according to the presence or absence of postoperative complications are summarized in Table 6.

**TABLE 5:** Presence of complication by type of surgical approach.

Complication	Open approach (n = 22)	Laparoscopic approach (n = 62)
Without complication	13 (59.0)	48 (77.4)
Operative site infection	5 (22.7)	4 (6.4)
Metabolic	0 (0)	1 (4.5)
Cardiopulmonary	1 (4.5)	3 (4.8)
Anastomotic dehiscence	1 (4.5)	1 (1.6)
Perforation	1 (4.5)	3 (4.8)
Hemorrhage	1 (4.5)	1 (4.5)
Other	0 (0)	1 (1.6)

**TABLE 6:** Distribution of clinically important variables according to the presence of complication

Variable	Complication, *N* (%) / x̄ ± SD	Without complication, *N* (%) / *x̄* ± SD
Age	58.7 ± 16.7	59.85 ± 17.45
Hospital stay	8.1 ± 4.3	5.29 ± 4.23
*Preoperative hemoglobin*		
<9	3 (3.5)	5 (5.9)
9–11	9 (10.7)	24 (28.5)
>11	11 (13.1)	32 (38.0)
*Preoperative albumin*		
2–2.5	1 (1.1)	2 (2.3)
2.6–3	3 (3.5)	9 (10.7)
3.1–3.5	12 (14.2)	35 (41.6)
>3.5	7 (8.3)	15 (17.8)
*Stage*		
0	3 (3.5)	0 (0)
I	9 (10.7)	1 (1.1)
IIA	5 (5.9)	1 (1.1)
IIB	6 (7.1)	3 (3.5)
IIC	6 (7.1)	4 (4.7)
IIIA	7 (8.3)	2 (2.3)
IIIB	10 (11.9)	3 (3.5)
IIIC	4 (4.7)	7 (8.3)
IVA	7 (8.3)	1 (1.1)
IVB	4 (4.7)	1 (1.1)
*Surgical approach*		
Open	9 (10.7)	13 (15.4)
Laparoscopy	14 (16.6)	48 (57.1)
*Type of surgery*		
Right hemicolectomy	6 (7.1)	16 (19.0)
Left hemicolectomy	6 (7.1)	8 (9.5)
Sigmoidectomy	3 (3.5)	2 (2.3)
Transversectomy	2 (2.3)	0 (0)
Total colectomy	0 (0)	2 (2.3)
Abdominoperineal resection	2 (2.3)	1 (1.1)
Low anterior resection	2 (2.3)	12 (14.2)
Ultra‐low anterior resection	0 (0)	5 (5.9)
Colostomy	2 (2.3)	15 (17.8)

Bivariate analysis yielded significant associations between the presence of postoperative complication and other variables for tumor location, anatomic subtype, type of surgery, initiation of the oral route, length of hospitalization and neoadjuvant therapy. Based on these results, a Poisson regression was performed to determine PR associated with the presence of postoperative complications **(**Table 7 **)**. Rectal anatomical location subtype was associated with the prevalence of complications (PR 0.36; 95% CI 0.13–0.96, and *p* = 0.04). However, when adjusting for confounding variables, the association persisted (PR 4.06; 95% CI 1.11–5.49, and *p* < 0.001). Additionally, it was found that for each additional day of hospitalization, the risk of postoperative complication was increased (PR 1.06, 95% CI 1.01–1.12, p = 0.01); but this association was lost in the adjusted analysis.

**TABLE 7:** Multivariate analysis between anatomic subtype, intraoperative complication, hospitalization and postoperative CEA value, with postoperative outcome

Variable	Crude PR	95% CI	p‐value	Adjusted PR	95% CI	p‐value
Subtype (distal)	1.19	0.57–2.45	0.63	4.06	2.71–6.09	0.06
Subtype (rectum)	0.36	0.13–0.96	0.04*	4.06	1.11–5.49	0.01
Intraoperative complication	1.38	1.21–1.59	0.01*	2.46	0.5–3.20	0.01
Hospitalization	1.06	1.01–1.12	0.04*	1	0.47–2.11	1
Postoperative CEA >15	6	0.86–41.44	0.06	–	–	–

* Statistically significant.

## 4 DISCUSSION

This study describes the factors associated with surgical and oncological outcomes in patients with colorectal cancer from the Colombian Caribbean region. Specifically, it was found that in this population, the anatomical subsite of the tumor location, the presence of intraoperative complication and the stay in critical care, influence the prognosis of this disease. The finding of predominant involvement of the rectum and diagnosis at a moderate to advanced stage suggests that the prognosis of colorectal cancer is poor and the burden of disease caused is high, confirming that this is a relevant public health problem in the region.

The results of the present study show a prevalence of postoperative complications of 27.6%. The distribution with respect to sex, age and location of the lesion observed is similar to that reported in the literature.^15^ The preoperative and operative characteristics highlighted are traditionally associated with prognosis and incidence of perioperative complications. Bivariate analysis showed a relationship between tumor location, anatomic subtype, type of surgery, initiation of the oral route, hospitalization time and neoadjuvant therapy. Remarkably, none of the patients who received adjuvant or neoadjuvant therapy presented postoperative complications, which could indicate an improvement in the prognosis of these patients. However, this characteristic did not allow the inclusion of this variable in the multivariate analysis. In agreement with what has been reported in the literature, a significant association was observed between more days of hospitalization and an increase in the prevalence of postoperative complications, which is related to the colonization of the patient's skin with nosocomial microorganisms that complicate the management of infections.^16^ This becomes important considering that the most frequently reported complication was surgical site infection. Although risk factors related to surgical site infection such as approach^17^, ^18^ and nutritional status are commonly reported in the literature,^19^ no significant associations were found between the presence of complications and these variables. However, there are several reasons that could explain the high prevalence of complications: (1) this referral center is a university hospital, so medical students and residents also have direct contact in the surgical and postoperative management of the patient, the lack of compliance with protocols could influence the incidence of postoperative infections; (2) many patients underwent emergency surgery, which does not allow for adequate preoperative monitoring (e.g., metabolic or nutritional profile); (3) those patients who presented complications had more advanced stages of cancer, which in turn was associated with a higher risk of complications due to organ dysfunction; (4) being a public referral center, the vast majority of patients are low‐income, so that the nutritional status and unhygienic lifestyle, substantially influences the organic recovery. This should arouse the interest of academic surgeons to further evaluate which strategies would positively influence the outcomes of these patients, depending on racial, economic, stage, and other subgroups.

At the international level, multiple series have been published on trends and operative outcomes in patients undergoing surgery for colon and rectal cancer. A few years ago, Augestad et al^20^ reported international trends in rectal cancer management through a survey of colorectal surgeons, representing 123 centers worldwide. The results highlight that the majority of surgeons responding to the survey reported performing more than 50 proctectomies annually, 72% laparoscopically, 76% routinely use diverting colostomies for anastomotic protection, and 63% use enhanced recovery protocols.^20^ Davis et al^21^ published a study that used the database of patients hospitalized in the United States in the period between 2004 and 2012, finding that 1 265 684 patients hospitalized for colorectal cancer.^21^ Additionally, they found that with respect to the trend in surgical modalities, there was a 35.4% decrease in open surgeries and a 3.5‐fold increase in laparoscopic surgeries, and a 41.3‐fold increase in robotic surgeries. However, open surgery was the preferred modality (65.4%) followed by laparoscopy (31.2%) and robotic surgery (3.4%).^21^ They also found that laparoscopic and robotic surgery were associated with lower in‐hospital mortality, fewer complications, shorter length of stay, which may be related to the elective nature of the procedure and lower degrees of tumor malignancy. Finally, when excluding patients with advanced stages, they found that laparoscopic surgery continued to be associated with better outcomes and lower costs than open surgery, while robotic surgery was associated with higher costs without significant benefits in perioperative outcomes compared to laparoscopic surgery.^21^

In Colombia, several studies have been carried out in the main cities of the country, analyzing the incidence and mortality of all types of colon and rectal cancer. Authors have found an average crude incidence rate of colon cancer of 9.0 vs. a mortality of 8.5. These data are lower than those found in Colombian cities, such as Cali,^22^ Bucaramanga,^23^ Manizales,^24^ and Pasto.^25^ After an exhaustive review of the literature, only two Colombian studies were found describing the experience in terms of perioperative outcomes of patients who underwent surgery for colon cancer. The first, published by Agudelo et al^26^ in 2017, where results of 152 patients attended in a University Hospital in the city of Bogotá were described, obtaining smoking as the most frequent risk factor, abdominal pain as the most frequent symptom followed by lower digestive bleeding, 61% of patients operated by laparoscopy and anastomosis dehiscence in 4.6% of patients (89.5% of patients did not present any complication related to the surgical procedure).^26^ Another study published in 2018 by Domínguez‐Herrera and Sirtori‐Campo,^27^ characterized outcomes in patients with laparoscopic surgery with a total of 32 cases, showing a hospital length of stay between 5 and 6 days in 50% and a stay of less than or equal to 4 days in 43.7% of patients.^27^

An aspect to highlight about the behavior of the outcomes in the approach to colorectal cancer and the need to continuously evaluate this phenomenon, is the search for and actual calculation of the impact of certain factors related to epigenetics, such as ethnicity.^28^ Evidence suggests that the performance of screening tests and the evolution of new cases vary between regions, making it difficult to extrapolate with confidence all the results found in the literature.^28^ It has been observed that in rural and marginalized areas belonging to low‐ and middle‐income countries, where there is a considerable volume of population (mainly due to farming and livestock activities or armed conflict situations), most of whom are elderly, there are inequities in education and screening interventions, which would explain difficulties in access to and compliance with therapeutic regimens and strict follow‐up, which increases mortality.^29^, ^30^ Taking into account, the aggressive behavior of colorectal cancer observed in the population studied, it can be inferred, for example, that the population of the rural area with lower education and greater economic hardship, are late in presenting gastrointestinal symptoms suggestive of the presence of cancer, which would explain the frequency of late stages of diagnosis. Therefore, these findings could support that in similar populations, which also have a high prevalence of comorbidities and are also exposed to tropical diseases, personalized strategies should be designed to promote screening and early detection of cancer. Although this was not the case, future studies could investigate the specific impact of belonging to the rural area as a risk factor for worse prognosis of colorectal cancer and, for example, decide to modify the management algorithms in this population, to avoid losing strict follow‐up and progression to advanced stages. Another comment of relevance would be that knowing the behavior of cancer progression in this population, it is of vital importance to monitor them for the performance of molecular studies of advanced cancer, due to the staging found at the time of diagnosis, which tends to be advanced. This needs to be taken into account during the discussion of the results and their applicability in the real field.

The presentation of the disease and the decision of the type of surgical and oncological approach depend on the time of diagnosis or severity of the cancer manifestation; as reported by Franklyn et al^31^ on the frequency of emergency first contact presentation among undiagnosed cancer patients living in remote areas, compared to control groups (27.7% vs. 19.7%, *p* < 0.01).^31^ In this order of ideas, the results obtained in this study are congruent with the conditions under which the patients were evaluated, and differ slightly in some aspects, which are typical of the progress in public policies and programs for early detection and specialized management of colorectal cancer, established in high‐income countries.

## 5 LIMITATIONS

The limitations of the present study include its cross‐sectional and retrospective descriptive nature. This limits the extrapolation of results and interferes with the interpretation of these results. Additionally, during data collection, incomplete or partial reporting of data in the clinical history was observed, which is a reflection of the fractionation of patient care, probably in multiple institutions, which make latent the need for the application of an organized protocol that guarantees the highest quality in the care of patients with colorectal surgery to improve morbimortality and prognosis.

## 6 CONCLUSIONS

The anatomical subsite of the neoplasm location, the presence of intraoperative complications and the stay in intensive care may be associated with the surgical and oncological outcome of individuals with colon cancer from the Colombian Caribbean region. Adenocarcinoma is the most frequent type of cancer in this population, the rectum the most common location, and most cases are detected in moderate and advanced stages, although in the short‐term the incidence of complications is low.

## AUTHOR CONTRIBUTIONS

**Edgard Ernesto Vergara‐Dagobeth:** Conceptualization (equal); investigation (equal); supervision (equal); writing – original draft (equal); writing – review and editing (equal). **Gian Alberto Núñez Rojas:** Conceptualization (equal); formal analysis (equal); supervision (equal); writing – original draft (equal). **Juan Carlos Hoyos‐Valdelamar:** Investigation (equal); methodology (equal); software (equal); writing – original draft (equal). **Ivan David Lozada‐Martinez:** Conceptualization (equal); investigation (equal); writing – original draft (lead); writing – review and editing (equal). **Amileth Suarez‐Causado:** Investigation (equal); methodology (equal); writing – original draft (equal); writing – review and editing (equal).

## FUNDING INFORMATION

This study was financed by the research office of the Universidad de Cartagena, through the projects identified with acts 78 and 114.

## CONFLICT OF INTEREST

The authors declare no conflicts of interest.

## Data Availability

The data that support the findings of this study are available from thecorresponding author, Alexis Rafael Narvaez‐Rojas, upon reasonable request
